
JOURNAL INFORMATION
==============================
NLM Title Abbreviation: ScientificWorldJournal
Journal ID: ScientificWorldJournal
NLM Journal ID: 101131163
Journal ID: TSWJ

The Scientific World Journal

ISSN: 2356-6140
EISSN: 1537-744X
Publisher: Wiley

ARTICLE INFORMATION
==============================
PMCID: PMC5848708
PMID: 18836657
DOI: 10.1100/tsw.2008.110
Article ID: 491850
Article version: 1
Subjects: Directions in Science

A Special Key for Unlocking the Door to Targeted Therapies of Breast Cancer

Lindemann Kristina 1 *
Harbeck Nadia 2
Lengyel Ernst 3
Resau James H. 4
1 Department of Obstetrics and Gynaecology, Medical Faculty, University of Oslo, Division Akershus University Hospital, Norway
2 Frauenklinik (Department of Obstetrics and Gynaecology) Technical University Munich, Germany
3 The University of Chicago Medical Center, Division of Gynecologic Oncology, Chicago, USA
4 Van Andel Research Institute, Grand Rapids, MI, USA
*Kristina Lindemann: kristina.lindemann@ahus.noAcademic Editor: Martin Goette

Publication date: 2008
Electronic publication date: 2008 Sep 21
Volume: 8
First page: 905
Last page: 908
Received 2008 Jul 1; Revised 2008 Aug 7; Accepted 2008 Aug 11
Copyright: Copyright © 2008 Kristina Lindemann et al.
Copyright year: 2008
License: This is an open access article distributed under the Creative Commons Attribution License, which permits unrestricted use, distribution, and reproduction in any medium, provided the original work is properly cited.
License URL: https://creativecommons.org/licenses/by/3.0/

Keywords: c-Met, Her2-neu, DCIS, targeted therapy

==============================
Directions in Science
*TheScientificWorldJOURNAL* (2008) 8, 905–908
ISSN 1537-744X; DOI 10.1100/tsw.2008.110

TheScientificWorldJOURNAL
www.thescientificworld.com

### A Special Key for Unlocking the Door to Targeted Therapies of Breast Cancer

Kristina Lindemann[1,*], Nadia Harbeck[2], Ernst Lengyel[3], and James H. Resau[4]

*[1]Department of Obstetrics and Gynaecology, Medical Faculty, University of Oslo, Division Akershus University Hospital, Norway; [2]Frauenklinik (Department of Obstetrics and Gynaecology), Technical University Munich, Germany; [3]The University of Chicago Medical Center, Division of Gynecologic Oncology, Chicago; [4]Van Andel Research Institute, Grand Rapids, MI*

**E-mail:** kristina.lindemann@ahus.no, nadia.harbeck@lrz.tum.de, elengyel@babies.bsd.uchicago.edu, James.Resau@vai.org

*Received July 1, 2008; Revised August 7, 2008; Accepted August 11, 2008; Published September 21, 2008*

**KEYWORDS:** c-Met, Her2-neu, DCIS, targeted therapy

The best hope for cancer therapy is currently thought to be personalized medicine and targeted therapies. Proof of principle has been shown with the drugs Gleevec® (bcr-abl) for chronic myeloid leukemia (CML) and Herceptin® (Her2-neu) for breast cancer. Both of these target a receptor that sits on the outside of cancer cells, much like the lock that opens a door. Receptor tyrosine kinases have come to fruition as therapeutic targets in a variety of malignancies. In particular, c-Met (http://www.vai.org/met/) and its ligand HGF/SF have gained considerable interest because of their role in growth, morphogenesis, and motility, as they work together to transform normal cells into malignant cells. The processes of cell motility, invasion, metastasis, epithelial to mesenchymal transition, angiogenesis, and tissue regeneration all involve c-Met and HGF/SF[1]. In breast cancer, their deregulation promotes cancer progression and prognosis[2,3,4,5,6].

Clinicians have become increasingly better at recognizing patients at risk for breast cancer (BRCA1, etc.), wide-spread screening programs improve early diagnosis, and instrumentation enables clinicians to sample these early and small lesions. Thus, what used to be a disease of invasive cancer is now a disease of an earlier and more difficult to define biologically *in situ* stage. Patients and clinicians need to know what is an objective and measurable feature in order to define the biological potential of these early and precursor lesions, and what will be a therapy particular to their lesion.

Ductal carcinoma *in situ* (DCIS) of the breast is the immediate precursor lesion of invasive breast cancer and therapeutic strategies have been more aggressive due to high recurrence rates. There is a strong need to direct therapy in a more precise matter and here c-Met is an intriguing target. Deregulated activation of the c-Met pathway in transformed cells has led to the development of multiple approaches for targeted therapies, and a variety of c-Met pathway inhibitors have been identified that not only target c-Met itself, but also disrupt the interaction of c-Met with its natural ligand HGF/SF. In particular, those breast cancers that are hormone receptor negative and Her2-neu negative (so called triple negatives) are becoming more prevalent, and there is no effective targeted therapy for them short of invasive surgery

*Corresponding author.
©2008 with author.
Published by TheScientificWorld; www.thescientificworld.com and nonspecific radiation as well as toxic chemotherapy. It is predicted that molecularly targeted therapy against c-Met will lead to dramatic inhibition of cancer growth and metastasis.

We recently published a study in *Histopathology* on the role of both c-Met, its ligand HGF/SF, and Her2-neu in DCIS of the breast[7]. Analysis of c-Met expression identified highly aggressive lesions, independent of Her2-neu expression. Both, up- and down-regulation of c-Met expression compared to surrounding normal tissue characterized a phenotype with unfavorable histopathology, such as Van Nuys (VN) Grade 3. DCIS cases classified as VN3 are considered to be high-grade lesions and are associated with a greater risk of recurrence[8]. We therefore believe that c-Met may be the key to understand DCIS aggressiveness and malignant progression, as well as a marker for targeted therapy in *in situ* carcinoma.

In breast cancer, c-Met overexpression is associated with tumor progression and poor survival. Its prognostic value has been demonstrated in early-stage patients with negative lymph nodes[9,10]. There are only a few studies on c-Met expression in DCIS, but they may elucidate the role of c-Met-HGF/SF signaling in early tumor progression. Jin et al.[11] observed significant stronger staining for c-Met and HGF/SF in DCIS than in benign hyperplasia, yet lower levels than in infiltrating carcinomas. Götte et al.[12] found c-Met overexpression more frequently in a subgroup of pure DCIS than when associated with a coexistent invasive carcinoma. This may contradict another finding of the study that c-Met exhibits a positive correlation with features of aggressiveness, such as HER2-neu overexpression, and may rather be indicative of the increased angiogenic activity in pure DCIS. Progressive loss of E-cadherin expression and a progressive increase in c-Met expression were observed with increasing dedifferentiation and higher metastatic potential. The prognostic impact of Her2-neu overexpression in invasive breast cancer has been widely demonstrated[13,14,15]. Its overexpression is even more frequently observed in DCIS than in invasive tumors[16,17], but its role in the progression to invasive disease is not completely understood. As Her2-neu overexpression may not play a key role in the progression of DCIS to invasive carcinoma[18], the investigation of c-Met expression may contribute to the understanding of their role in tumor development and progression.

In our study, the expression of both c-Met and Her2-neu in DCIS of the breast was analyzed for the first time using two independent immunocytochemical staining techniques. Most results obtained by conventional immunohistochemistry were confirmed by confocal immunofluorescent analysis. We did not observe a significant correlation between c-Met and Her2-neu overexpression, indicating that c-Met expression in DCIS is independent of Her2-neu overexpression. Analysis of c-Met expression identified highly aggressive lesions with an imbalance in c-Met expression between tumor lesion and surrounding tissue. This is consistent with the hypothesis that tumors with an imbalance between c-Met expression in tumor vs. normal tissue are less well differentiated and have a higher proliferative activity[19]. Recently, Shattuck et al.[20] even showed how breast cancer cells use c-Met receptor signaling as a method to overcome trastuzumab resistance. There were no obvious signs of intense tumor-stroma interaction with regard to HGF/SF expression in our study. The fact that HGF/SF is the essential ligand for metastasis and that paracrine secretion might contribute to an invasive behavior leads to the assumption that this missing secretion contributes to the inability of the lesions to degrade the basement membranes. Our results suggest that the balance between c-Met expression in the tumor and adjacent normal tissue, together with a paracrine HGF/SF secretion, could be the key to understand the invasive potential in DCIS.

The overexpression, mutation, and amplification of c-Met in tumor cells underline its pleiotropic functions and hint at c-Met as an ideal target in clinical therapeutics. This has led to the development of multiple approaches for targeted therapies both by inhibition of c-Met or c-Met peptides[21,22,23,24], inhibition of HGF/SF activation of c-Met[25], or targeting c-Met expression at RNA level[26,27]. The reported study suggests biologic features, such as c-Met expression, which may help to identify highly aggressive DCIS lesions, which require larger excision margins, more extensive local and medical treatment, or a different follow-up.

Further studies on the role of c-Met and HGF/SF in the transition from DCIS to invasive carcinoma are needed in order to understand which factors will influence this balance, and how c-Met and HGF/SF are regulated during tumor progression. Efficient therapeutic approaches may need to target simultaneously multiple molecules that are relevant to this early step of breast cancer progression. In the future, we hope to be able to determine if c-Met-HGF/SF inhibition is an effective cancer treatment for DCIS as it is expected to be an important signaling target in a large number of malignancies.

### REFERENCES

1. Weidner, K.M., Sachs, M., and Birchmeier, W. (1993) The met receptor tyrosine kinase transduces motility, proliferation, and morphogenic signals of scatter factor/hepatocyte growth factor in epithelial cells. *J. Cell. Biol.* **121**, 145–154.
2. Yamashita, J., Ogawa, M., Yamashita, S., et al. (1994) Immunoreactive hepatocyte growth factor is a strong and independent predictor of recurrence and survival in human breast cancer. *Cancer Res.* **54**, 1630–1633.
3. Jeffers, M., Rong, S., and Vande Woude, G.F. (1996) Hepatocyte growth factor/scatter factor-Met signaling in tumorigenicity and invasion/metastasis. *J. Mol. Med.* **74**, 505–513.
4. Comoglio, P.M. and Trusolino, L. (2002) Series introduction: invasive growth: from development to metastasis. *J. Clin. Invest.* **109**, 857–862.
5. Kang, J.Y., Dolled-Filhart, M., Ocal, I.T., Singh, B., Lin, C.Y., Dickson, R.B., Rimm, D.L., and Camp, R.L. (2003) Tissue microarray analysis of hepatocyte growth factor/met pathway components reveals a role for met, matriptase, and hepatocyte growth factor activator inhibitor 1 in the progression of node-negative breast cancer. *Cancer Res.* **63**, 1101–1105.
6. Lengyel, E., Prechtel, D., Resau, J.H., Gauger, K., Welk, A., Lindemann, K., Salanti, G., Richter, T., Knudsen, B., Vande Woude, G.F., and Harbeck, N. (2005) C-Met overexpression in node-positive breast cancer identifies patients with poor clinical outcome independent of Her2/neu. *Int. J. Cancer* **113**, 678–682.
7. Lindemann, K., Resau, J.H., Nährig, J., Kort, E., Leeser, B., Annecke, K., Welk, A., Schäfer, J., Vande Woude, G.F., Lengyel, E., and Harbeck, N. (2007) Differential expression of c-Met, its ligand HGF/SF and HER2/neu in DCIS and adjacent normal breast tissue. *Histopathology* **51**, 54–62.
8. Silverstein, M.J., Lagios, M.D., Craig, P.H., Waisman, J.R., Lewinsky, B.S., Colburn, W.J., et al. (1996) A prognostic index for ductal carcinoma in situ of the breast. *Cancer* **77**, 2267–2274.
9. Camp, R.L., Rimm, E.B., and Rimm D.L. (1999) *Met* expression is associated with poor outcome in patients with axillary lymph node negative breast carcinoma. *Cancer* **86**, 2259–2265.
10. Ocal, I.D., Dolled-Filhart, M., D'Aquila, T.G., Camp, R.L., and Rimm, D.L. (2003) Tissue microarray based studies of patients with lymph node negative breast carcinoma show that met expression is associated with worse outcome but it is not correlated with epidermal growth factor family receptors. *Cancer* **97**, 1841–1848.
11. Jin, L., Fuchs, A., Schnitt, S.J., Yao, Y., Joseph, A., Lamszus, K., Park, M., Goldberg, I.D., and Rosen, E.M. (1997) Expression of scatter factor and c-*met* receptor in benign and malignant breast tissue. *Cancer* **79**, 749–760.
12. Götte, M., Kersting, C., Radke, I., Kiesel, L., and Wülfing, P. (2007) An expression signature of syndecan-1 (CD138), E-cadherin and c-met is associated with factors of angiogenesis and lymphangiogenesis in ductal breast carcinoma in situ. *Breast Cancer Res.* **9**, R8.
13. Borg, A., Tandon, A.K., Sigurdsson, H., Clark, G.M., Fernö, M., Fuqua, S.A., Killander, D., and McGuire, W.L. (1990) Her-2/*neu* amplification predicts poor survival in node-positive breast cancer. *Cancer Res.* **50**, 4332–4337.
14. Ferrero-Pous, M., Hacene, K., Bouchet, C., Le Doussal, V., Tubiana-Hulin, M., and Spyratos, F. (2000) Relationship between c-erbB-2 and other tumor characteristics in breast cancer prognosis. Clin. *Cancer Res.* **6**, 4745–4754.
15. Menard, S., Fortis, S., Castiglioni, F., Agresti, R., and Balsari, A. (2001) HER2 as a prognostic factor in breast cancer. *Oncology* **61**, 67–72.
16. van de Vijver, M.J., Peterse, J.L., Mooi, W.J., Wisman, P., Lomans, J., Dalesio, O., and Nusse, R. (1988) Neu-protein overexpression in breast cancer. Association with comedo-type ductal carcinoma in situ and limited prognostic value in stage II breast cancer. *N. Engl. J. Med.* **319**, 1239–1245.
17. Allred, D.C., Clark, G.M., Tandon, A.K., Molina, R., Tormey, D.C., Osborne, C.K., Gilchrist, K.W., Mansour, E.G., Abeloff, M., Eudey, L., et al. (1992) Her-2/neu in node-negative breast cancer: prognostic significance of overexpression influenced by the presence of in situ carcinoma. *J. Clin. Oncol.* **10**, 599–605
18. Latta, E.K., Tjan, S., Parkes, R.K., and O'Malley, F.P. (2002) The role of Her2/neu overexpression/amplification in the progression of ductal carcinoma in situ to invasive carcinoma of the breast. *Mod. Pathol.* **15**, 1318–1325.
19. Altstock, R.T., Stein, G.Y., Resau, J.H., and Tsarfaty, I. (2000) Algorithms for quantification of protein expression variation in normal versus tumor tissue as a prognostic factor in cancer: Met oncogene expression, and breast cancer as a model. *Cytometry* **41**, 155–165.
20. Shattuck, D.L., Miller, J.K., Carraway, K.L., 3rd, and Sweeney, C. (2008) Met receptor contributes to trastuzumab resistance of Her2-overexpressing breast cancer cells. *Cancer Res.* **68**, 1471–1477.
21. Morotti, A., Mila, S., Accornero, P., Tagliabue, E., and Ponzetto, C. (2002) K252a inhibits the oncogenic properties of Met, the HGF receptor. *Oncogene* **21**, 4885–4893.
22. Christensen, J.G., Schreck, R., Burrows, J., Kuruganti, P., Chan, E., Le, P., Chen, J., Wang, X., Ruslim, L., Blake, R., Lipson, K.E., Ramphal, J., Do, S., Cui, J.J., Cherrington, J.M., and Mendel, D.B. (2003) A selective small molecule inhibitor of c-Met kinase inhibits c-Met-dependent phenotypes in vitro and exhibits cytoreductive anti-tumor activity in vivo. *Cancer Res.* **63**, 7345–7355.

23. Sattler, M., Pride, Y.B., Ma, P., Gramlich, J.L., Chu, S.C., Quinnan, L.A., Shirazian, S., Liang, C., Podar, K., Christensen, J.G., and Salgia, R. (2003) A novel small molecule met inhibitor induces apoptosis in cells transformed by the oncogenic TPR-MET tyrosine kinase. *Cancer Res.* **63**, 5462–5469.
24. Cassinelli, G., Lanzi, C., Petrangolini, G., Tortoreto, M., Pratesi, G., Cuccuru, G., Laccabue, D., Supino, R., Belluco, S., Favini, E., Poletti, A., and Zunino, F. (2006) Inhibition of c-Met and prevention of spontaneous metastatic spreading by the 2-indolinone RPI-1. *Mol.. Cancer. Ther.* **5**, 2388–2397.
25. Bardelli, A., Longati, P., Williams, T.A., Benvenuti, S., and Comoglio, P.M. (1999) A peptide representing the carboxyl-terminal tail of the met receptor inhibits kinase activity and invasive growth. *J. Biol. Chem.* **274**, 29274–29281.
26. Herynk, M.H., Stoeltzing, O., Reinmuth, N., Parikh, N.U., Abounader, R., Laterra, J., Radinsky, R., Ellis, L.M., and Gallick, G.E. (2003) Down-regulation of c-Met inhibits growth in the liver of human colorectal carcinoma cells. *Cancer Res.* **63**, 2990–2996.
27. Shinomiya, N., Gao, C.F., Xie, Q., Gustafson, M., Waters, D.J., Zhang, Y.W., and Vande Woude, G.F. (2004) RNA interference reveals that ligand-independent met activity is required for tumor cell signaling and survival. *Cancer Res.* **64**, 7962–7970.

**This article should be cited as follows:**

Lindemann, K., Harbeck, N., Lengyel, E., and Resau, J.H. (2008) A special key for unlocking the door to targeted therapies of breast cancer. *TheScientificWorldJOURNAL* **8**, 905–908. DOI 10.1100/tsw.2008.110.
